# Supplementary material for: Virtual Screening of Novel 24-Dehydroxysterol Reductase (DHCR24) Inhibitors and the Biological Evaluation of Irbesartan in Cholesterol-Lowering Effect
Source: Molecules. 2023 Mar 14;28(6):2643. doi: 10.3390/molecules28062643 (PMC10053925; doi:10.3390/molecules28062643)
Supplement: Supplementary file 1 [file molecules-28-02643-s001.zip › molecules-2225580-supplementary.pdf]

Supplementary Materials

Virtual Screening of Novel 24-Dehydroxysterol Reductase (*DHCR24*) Inhibitors and the Biological Evaluation of Irbesartan in Cholesterol-Lowering Effect

Haozhen Wang [1,†], Ziyin Lu [1,†], Yang Li [1], Ting Liu [1], Linlin Zhao [1], Tianqi Gao [1], Xiuli Lu [1,*] and Bing Gao [2,*]

[1] The School of Life Science, Liaoning University, Chongshanzhong-Lu No.66, Shenyang 110036, China
[2] School of Basic Medical Sciences, Shenyang Medical College, Shenyang 110034, China
* Correspondence: luxiuli@lnu.edu.cn (X.L.); gaobingdr@hotmail.com (G.B.); Tel.: +86-24-62202232 (X.L.); +86-24-62215664 (B.G.)
† These authors contributed equally to this work.

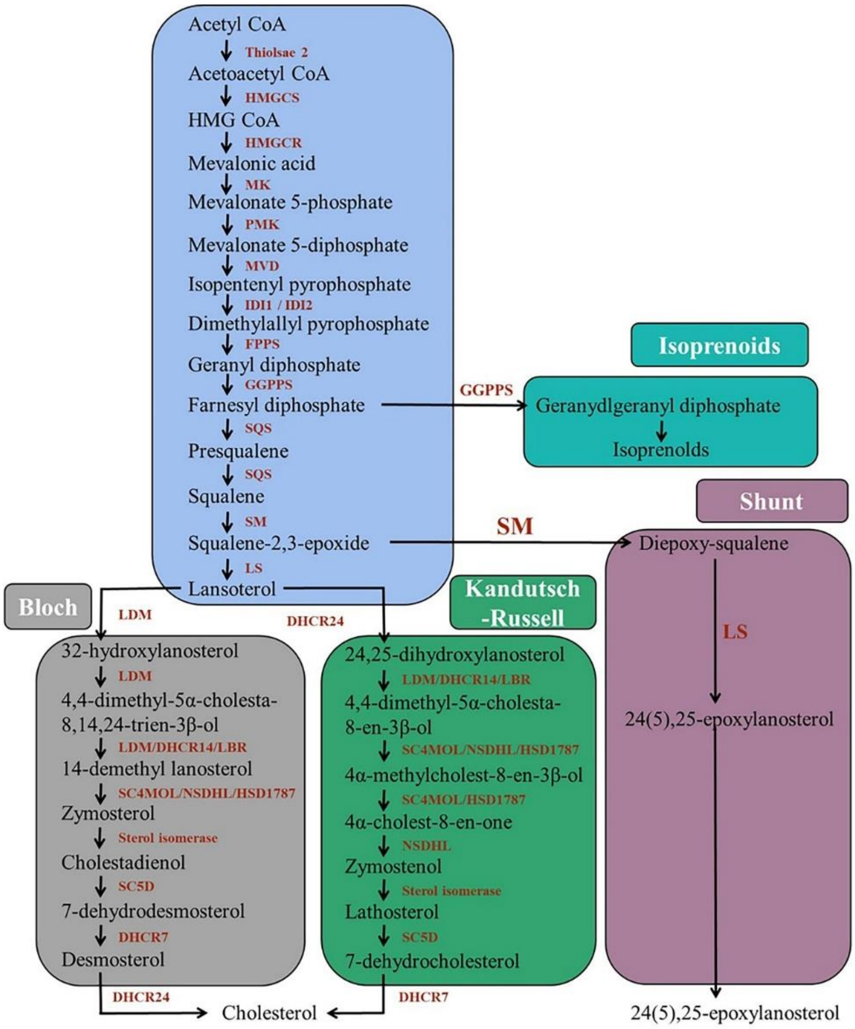

Figure S1. Cholesterol synthesis pathway.
